# Evaluation of Systematic Assessment of Asthma-Like Symptoms and Tobacco Smoke Exposure in Early Childhood by Well-Child Professionals: A Randomised Trial

**DOI:** 10.1371/journal.pone.0090982

**Published:** 2014-03-13

**Authors:** Esther Hafkamp-de Groen, Ralf J. P. van der Valk, Ashna D. Mohangoo, Johannes C. van der Wouden, Liesbeth Duijts, Vincent W. Jaddoe, Albert Hofman, Harry J. de Koning, Johan C. de Jongste, Hein Raat

**Affiliations:** 1 The Generation R Study Group, Erasmus Medical Center, Rotterdam, The Netherlands; 2 Department of Public Health, Erasmus Medical Center, Rotterdam, The Netherlands; 3 Department of Paediatrics, Division of Respiratory Medicine, Erasmus Medical Center-Sophia Children’s Hospital, Rotterdam, The Netherlands; 4 Department of Epidemiology, Erasmus Medical Center, Rotterdam, The Netherlands; 5 TNO, Netherlands Organisation for Applied Scientific Research, Department of Child Health, Leiden, The Netherlands; 6 Department of General Practice and Elderly Care Medicine, EMGO, VU University Medical Center, Amsterdam, The Netherlands; 7 Department of Paediatrics, Division of Neonatology, Erasmus Medical Center-Sophia Children’s Hospital, Rotterdam, The Netherlands; 8 Department of Paediatrics, Erasmus Medical Center-Sophia Children’s Hospital, Rotterdam, The Netherlands; Universität Bochum, Germany

## Abstract

**Objectives:**

This study aimed to evaluate the effectiveness of systematic assessment of asthma-like symptoms and environmental tobacco smoke (ETS) exposure during regular preventive well-child visits between age 1 and 4 years by well-child professionals.

**Methods:**

Sixteen well-child centres in Rotterdam, the Netherlands, were randomised into 8 centres where the brief assessment form regarding asthma-like symptoms and ETS exposure was used and 8 centres that applied usual care. 3596 and 4179 children (born between April 2002 and January 2006) and their parents visited the intervention and control centres, respectively. At child’s age 6 years, physician-diagnosed asthma ever, wheezing, fractional exhaled nitric oxide (FeNO), airway resistance (Rint), health-related quality of life (HRQOL) and ETS exposure at home ever were measured. Linear mixed models were applied.

**Results:**

No differences in asthma, wheezing, FeNO, Rint or HRQOL measurements between intervention and control group were found using multilevel regression in an intention-to-treat analysis (p>0.05). Children of whom the parents were interviewed by using the brief assessment form at the intervention well-child centres had a decreased risk on ETS exposure at home ever, compared to children who visited the control well-child centres, in an explorative per-protocol analysis (aOR = 0.71, 95% CI:0.59–0.87).

**Conclusions:**

Systematic assessment and counselling of asthma-like symptoms and ETS exposure in early childhood by well-child care professionals using a brief assessment form was not effective in reducing the prevalence of physician-diagnosed asthma ever and wheezing, and did not improve FeNO, Rint or HRQOL at age 6 years. Our results hold some promise for interviewing parents and using information leaflets at well-child centres to reduce ETS exposure at home in preschool children.

**Trial Registration:**

Controlled-Trials.com ISRCTN15790308.

## Introduction

Asthma is a highly prevalent chronic condition associated with considerable morbidity, reduced health-related quality of life (HRQOL) and significant costs for public health [1], [2]. Interventions aimed at preventing childhood asthma are being developed and evaluated [3]–[9]. While the majority of asthma management education for parents occurs in the clinical setting, increasingly, multifaceted environmental interventions to decrease asthma-like symptoms are delivered by community health workers [7]. Previous studies identified positive outcomes associated with community health worker-delivered interventions, including decreased asthma-like symptoms [7].

In the Netherlands, growth, development and health of all children (0–19 years) is monitored in a nationwide program with regular visits at set ages by well-child care physicians and nurses [10]. The nationwide program is offered free of charge by the government and participation is voluntary (attendance rate ca. 90%) [11]. The well-child care setting creates an opportunity for tailored prevention and promotion of healthy child development. During well-child visits, among other topics that are relevant at the developmental stage of the child, the well-child professionals (medical doctors and nurses) should pay attention to the presence of asthma-like symptoms. However, until now, no systematic assessment of the presence of asthma-like symptoms in early childhood by well-child professionals has been applied at well-child centres in the Netherlands. In the Netherlands, the nationwide well-child care program advises to interview parents regarding environmental tobacco smoke (ETS) exposure to preschool children [11]. However, information leaflets with regard to ETS exposure are not yet given routinely to parents of children aged 1 to 4 years who are exposed to ETS.

This study aimed to evaluate the effectiveness of systematic assessment of asthma-like symptoms and ETS exposure between age 1 and 4 years by well-child professionals. We hypothesised that systematic assessment of asthma-like symptoms and ETS exposure to parents of preschool children (and subsequent counselling such as providing information leaflets or arranging a referral when needed) reduces the prevalence of physician-diagnosed asthma ever and wheezing frequency, and improves fractional exhaled nitric oxide (FeNO, a biomarker of airway inflammation), airway resistance (Rint) and HRQOL measurements at age 6 years. In addition to the study protocol [12], we evaluated whether this approach resulted in a reduction of ETS exposure at home (‘ETS exposure at home ever’ measured at child age 6 years).

## Methods

### Ethics Statement

This study is embedded in the Generation R Study, a prospective population-based cohort [13], in collaboration with the regional well-child care organisation Centre for Youth and Family in Rotterdam. The Generation R Study was conducted in accordance with the guidelines proposed in the Declaration of Helsinki, and was approved by the Medical Ethical Committee of the Erasmus Medical Centre. All parents who participated in the Generation R Study provided written informed consent for the use of data regarding their child for research aimed at identifying factors influencing the health of young children. In this study, to evaluate the brief assessment form regarding asthma-like symptoms and ETS exposure applied by well-child professionals, we used data that were collected in the Generation R Study. We are prepared to make the data available upon request.

### Study Design

**Figure 1:**
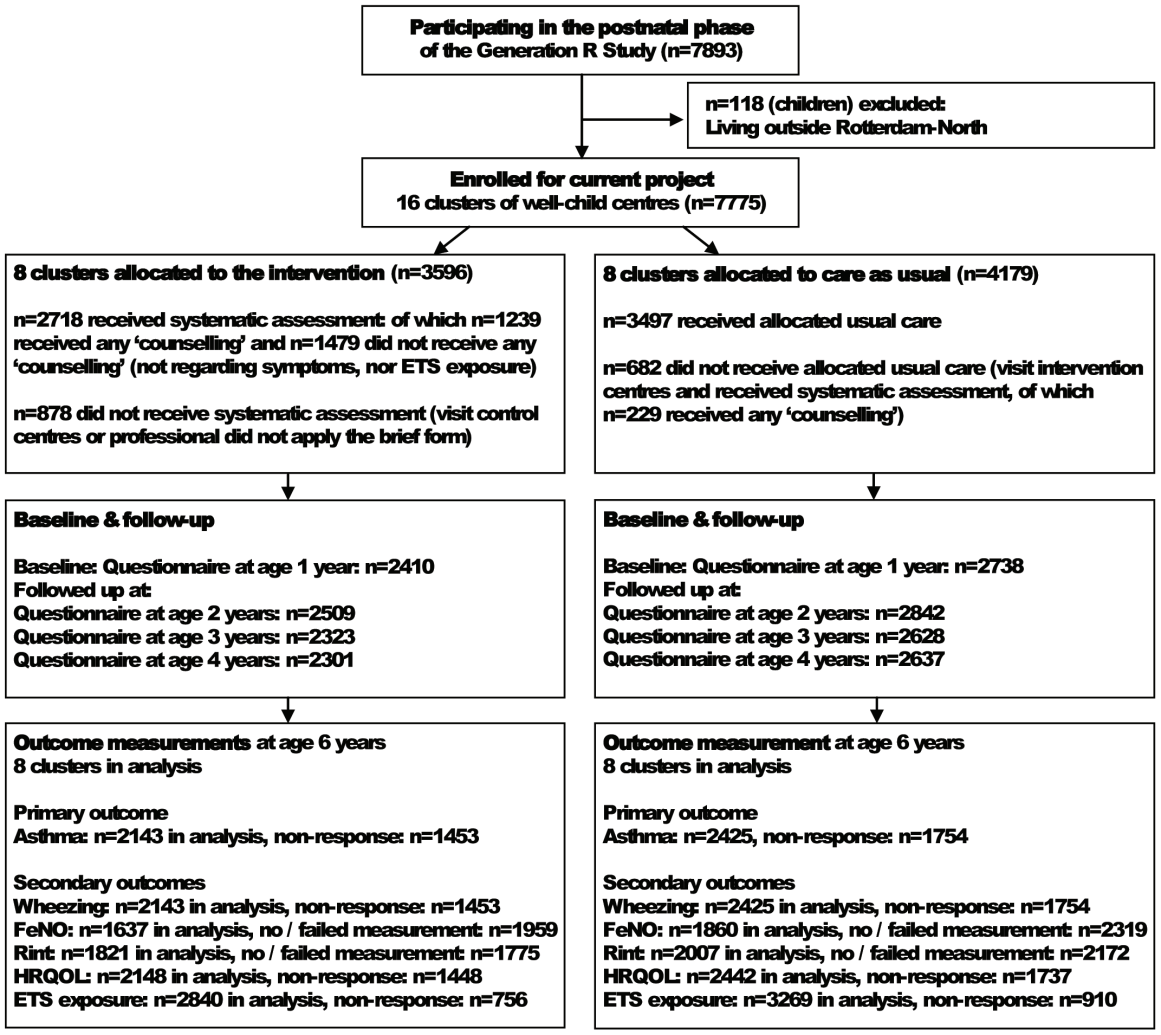
Flow of participants through the study. FeNO = fractional exhaled nitric oxide, HRQOL = health related quality of life, Rint = airway resistance, ETS = environmental tobacco smoke.

Details of our study design were published previously (see File S1) [12]. This study started in June 2005 and follow-up at age 6 years was completed in January 2012. In total, 7775 children (born between April 2002 and January 2006) entered the study (Fig. 1). Sixteen well-child centres that participated in the data collection of the Generation R Study were randomized into 8 well-child centres that applied the brief assessment form regarding asthma-like symptoms and ETS exposure at each regularly scheduled visit to the well-child centre between age 1 and 4 years, and 8 centres that applied usual care. First, the well-child centres were ranked (by researcher ADM) based on the socioeconomic status of their neighbourhood. Well-child centres in each subsequent couple in this list were randomly assigned to the intervention group (n = 8) or the control group (n = 8). Parents were not aware of the research condition they were allocated to. The protocol for this trial and supporting CONSORT checklist are available as supporting information; see Checklist S1 and Protocol S1.

### Intervention and Usual Care

When parent and child attended the well-child centre allocated to the intervention group, the professionals used a brief assessment form regarding asthma-like symptoms and ETS exposure during the regular visits at age 14, 24, 36 and 45 months. Details of this form were published previously [12]. In summary, with regard to asthma-like symptoms the brief form included items on wheezing, and shortness of breath or dyspnea. Furthermore, the form included an item that assessed whether the child had been exposed to ETS during the past year (no, yes-sometimes, yes-on a regular basis, yes-often or daily, unknown).

When parents reported that their child had at least 3 episodes of any asthma-like symptoms during the past 12 months and at least 1 episode of asthma-like symptoms in the past 4 weeks, the well-child professionals could provide them with a leaflet with information about asthma. If the child had been free of asthma-like symptoms during the past 4 weeks, the well-child professionals could advise a visit to the general practitioner should the child’s asthma-like symptoms return. When parents reported that their child had at least 3 episodes of asthma-like symptoms during the past 12 months, of which at least 1 in the past 4 weeks, and the child had not yet been treated by the general practitioner or paediatrician in the past 4 weeks, the well-child professionals could refer to the asthma nurse and/or general practitioner. If the child had already been treated by the general practitioner or paediatrician in the past 4 weeks, the well-child professionals could refer to the asthma nurse.

If the child had been exposed to ETS (sometimes, on a regular basis, often or daily), the well-child professional could discuss health risks of ETS exposure to preschool children (health risks), and discuss whether parents could be motivated and prepared to stop ETS exposure to their child (house rules) and provide them with an information leaflet about preventing their child from exposure to ETS. The well-child professionals from the intervention centres were informed during a two-hour session about the intervention.

The control centres applied current routine practice, addressing the presence of general health symptoms during the regular well-child visits and ETS exposure (at least at age 18 months) [11]. However, no specific, systematic assessment of the presence of asthma-like symptoms and ETS exposure by the use of a brief form was performed by the well-child professionals in the control group.

### Primary and Secondary Outcomes

Data from parents were collected in the Generation R Study by postal questionnaires at enrolment, and at the first, 2^nd^, 3^rd^, 4^th^ and 6^th^ year of life. Response rates for these questionnaires were 71%, 76%, 72%, 73% and 68%, respectively. The primary outcome measure was physician-diagnosed asthma ever, obtained by a parent-reported questionnaire at age 6 years.

Secondary outcomes were current wheezing frequency (as reported by parents), FeNO, Rint and HRQOL as reported by parents. Reducing ETS exposure to preschool children was one of the objectives of counselling following systematic assessment of ETS. Therefore, in addition to the proposed outcomes [12], we evaluated at age 6 years whether the intervention had reduced ETS exposure at home ever (as reported by parents).

Wheezing frequency (never, 1–3 episodes, ≥4 episodes) in the past 12 months was assessed using a parent-reported question from the International Study of Asthma and Allergies in Childhood (ISAAC) [14].

FeNO was measured according to American Thoracic Society guidelines [15] at age 6 years at the research centre (NIOX chemiluminescence analyser, Aerocrine AB, Solna, Sweden). Statistical analyses were additionally adjusted for technique to take into account computer-calculated and researcher-observed FeNO values. FeNO was normalized by ^e^log transformation.

At age 6 years, Rint (Micro Rint, MicroMedical, Rochester, Kent, UK) was measured at the research centre during tidal breathing, with occlusion of the airway at tidal peak expiratory flow. Median values for at least 5 acceptable Rint measurements were calculated and used to calculate Z-scores, additionally adjusted for median variation of the study period [16], [17].

The CHQ-PF28 in the parent-reported questionnaire was used to measure HRQOL of the child at age 6 years [18]. Based on 28 items, the CHQ-PF28 measures the HRQOL of children and their families across 13 scales [19], [20]. The following eight multi-item scales measure the child’s HRQOL*: Physical functioning*, *Role functioning: emotional*, *Role functioning: physical*, *Bodily pain*, *General behaviour*, *Mental health*, *Self-esteem*, *General health perceptions*. These multi-item scales were summarised into a *Physical summary measure* and a *Psychosocial summary measure*. Furthermore we used the *Change in health* item. The impact of the child’s health on the caregiver’s and family’s HRQOL was measured across the remaining four multi-item scales: *Parental impact: emotional*, *Parental impact: time*, *Family cohesion* and *Family activities*. All scale measures were transformed to scores ranging from 0 to 100. Lower scores correspond to lower HRQOL. Summary measures were standardised with a mean of 50 and standard deviation of 10 to reflect general US population norms for children [19], [20].

The outcome ‘ETS exposure at home ever (yes, no)’ at age 6 years was defined and based on parent-reported questionnaires at age 2, 3 and 6 years, using the question: ‘Do people smoke occasionally at home? (yes, no)’. ‘ETS exposure at home ever’ at age 6 years was scored ‘yes’ if there was ETS exposure at home at age 2 or 3 or 6 years.

### Covariates

We used information collected in the Generation R Study on maternal characteristics (educational level, net household income, ethnicity, single motherhood and history of asthma or atopy) for the intervention and control group. Information about the highest attained maternal educational level (low, moderate, high), maternal ethnicity (Dutch, other western, non-western) and single motherhood (yes, no) and maternal history of asthma or atopy (yes, no) were obtained at enrolment by questionnaires. Maternal educational level and maternal ethnicity were defined according to the classification of Statistics Netherlands [21], [22]. Data on household income (<€1600/month, ≥€1600/month) was obtained at the child’s age of 3 years, using the 2005 monthly general labour income as the cut-off point [23]. Information on child’s gender (boy, girl), gestational age at birth (weeks) and birth weight (grams), were obtained from medical records. We used information collected in the Generation R Study on child’s characteristics that were established using parent-reported questionnaires which included: ETS exposure at home (yes, no) (reported during pregnancy) [24]; breastfeeding ever at age 0–6 months (yes, no); keeping pets (yes, no) at the 1^st^ year of life; respiratory tract infections (yes, no) and wheezing (yes, no) at the 1^st^ year of life.

### Statistical Analyses

Baseline data for the intervention and control group were described using descriptive statistics, which were tested for differences using multinomial regression adjusted for randomisation stratum (cluster). All participants were analysed according to the “intention-to-treat” principle.

The prevalence of ETS exposure at home before (fetal life to age 6 months), during (at age 14–45 months) and after (at age 6 years) the study period was described. *P* values for differences in the prevalence of ‘ETS exposure at home’ between intervention and control group were calculated by means of the Chi-square test. Although not according to the study protocol, several children participating in the control group also visited the intervention centres and assessment of asthma-like symptoms and ETS exposure by a brief form was applied to a part of the parents of these children. Contamination of intervention and control condition may possibly also have occurred by moving to another neighbourhood in the city and visiting another well-child centre. Because this contamination may have reduced the differences in results between intervention and control group, we amended the study protocol [12] and in addition to the intention-to-treat analyses we performed a per-protocol analysis. In the per-protocol analysis we included children who were allocated to the intervention group and also received the allocated intervention (n = 2718). In the control group only children were included when they were allocated to the control group and received usual care (n = 3497) (see Fig. 1). Outcomes at age 6 years were predicted with a model using two predictors: research condition (intervention or usual care) and baseline value of the outcome variable [25], [26].

To prevent bias associated with attrition, missing data at baseline and missing outcomes were multiple imputed (10 imputed datasets) on the basis of the correlation between each variable with missing values and other parental and child characteristics [27] to reduce bias and improve efficiency [28]. Regression analyses were performed in the original data and after the multiple imputation procedure. Since we found similar effect estimates (with and without multiple imputation) the final results in our paper are presented as effect estimates with its 95% Confidence Intervals (95%CI) with adjustment for randomisation stratum, derived from the original (unimputed) data. Multilevel regression analyses were applied to allow for dependency between the individual measurements within the 16 randomised well-child centres. (the GENLINMIXED procedure in SPSS and PROC GLIMMIX procedure in SAS) [29], [30]. We considered two levels: the cluster level (well-child center) and the individual(child) level. In the final model, we used the default covariance structure in the multilevel regression analysis in SPSS. The difference between intervention and control group on the categorical outcomes ‘physician-diagnosed asthma ever (yes/no)’ and ‘ETS exposure at home (yes/no)’ were studied using the ‘binomial’ distribution and link = logit. The difference between intervention and control group on the categorical outcome ‘Wheezing frequency (never, 1–3 times/year, >3 times/year)’ was studied using the ‘multinomial’ distribution and link = logit. The differences between intervention and control group on the health-related quality of life scales were studied using the ‘poisson’ distribution and link = log. The differences between intervention and control group on the outcomes FeNO and Rint were studied using the ‘normal’ distribution and link = identity. FeNO was normalized by ^e^log transformation.

Potential effect modification of socio-demographic characteristics and baseline values of the outcomes on the association between the research condition (intervention or care as usual group) and the outcomes was explored. First, we fit a multinomial regression model with randomisation stratum and baseline values of the outcome. Second, we added socio-demographic characteristics (child’s gender and maternal ethnic background and educational level) and baseline values of the outcomes as an interaction separately [12], [31], [32]. The interaction terms were evaluated at p<0.10 level [33].

Random treatment allocation ensures that intervention status will not be confounded with either measured or unmeasured baseline characteristics [34]. Therefore, the effect of the intervention on outcomes was estimated by comparing outcomes between the intervention and control group, only adjusted for randomisation stratum and baseline prevalence of the outcomes.

It should be considered that given multiple comparisons, there is an 1-in-20 chance of a false association for each comparison (Type I error at p = 0.05) [35]. Bonferroni correction was applied to correct for multiple testing (P = 0.05/number of comparisons) [35].

In addition, a process evaluation of the intervention was performed. The study is reported according to the CONSORT standards for reporting RCTs [30], [36]. Analyses were performed using the Statistical Package for Social Sciences (SPSS) version 20.0 for Windows (SPSS Inc, Chicago, IL, USA) and SAS 9.2 (SAS Institute, Cary, NC, USA).

## Results

### Recruitment

There were 8 intervention and 8 control well-child centres, involving 3596 and 4179 children (and their parents) visiting these well-child centres, respectively. The different rates of participation of the children in the different elements of the study are shown in the flow diagram (Fig. 1).

Table 1 summarizes the baseline characteristics of the study population, stratified by intervention and control group. At baseline, no differences were found between the characteristics of the intervention and control group, after adjustment for randomisation stratum (p>0.05).

**Table 1:** Baseline characteristics by allocation group (n = 7775).

		Total	Intervention	Care as usual	*P* value*
	Missing	N = 7775 16 clusters	n = 3596 (46.3%) 8 clusters	n = 4179 (53.7%) 8 clusters	
**Maternal characteristics**					
Educational level	732 (9.4)				
Low		1610 (22.9)	717 (21.8)	893 (23.8)	0.96
Middle		2081 (29.5)	954 (29.0)	1127 (30.0)	
High		3352 (47.6)	1617 (49.2)	1735 (46.2)	
Net household income	2101 (27.0)				
<1600 €/month		1536 (27.1)	608 (23.6)	928 (29.9)	0.56
≥1600 €/month		4138 (72.9)	1966 (76.4)	2172 (70.1)	
Ethnicity	736 (9.5)				
Dutch		3817 (54.2)	1884 (57.4)	1933 (51.5)	0.48
Other western		1186 (16.8)	498 (15.2)	688 (18.3)	
Non-western		2036 (28.9)	900 (27.4)	1136 (30.2)	
Single motherhood (yes)	892 (11.5)	865 (12.6)	408 (12.7)	457 (12.4)	0.93
Smoking during pregnancy (yes)	1717 (22.1)	1510 (24.9)	679 (24.5)	831 (25.3)	0.40
History of asthma or atopy (yes)	1608 (20.7)	2402 (38.9)	1140 (39.1)	1262 (38.8)	0.80
**Child’s characteristics**					
Gender (male)	0 (0)	3920 (50.4)	1796 (49.9)	2124 (50.8)	0.44
Gestational age at birth	0 (0)				
<37 weeks		472 (6.1)	208 (5.8)	264 (6.3)	0.35
≥37 weeks		7303 (93.9)	3388 (94.2)	3915 (93.7)	
Birth weight (grams)	0 (0)				
<2500 grams		438 (5.6)	189 (5.3)	249 (6.0)	0.24
≥2500 grams		7337 (94.4)	3407 (94.7)	3930 (94.0)	
Breastfeeding ever (yes)	1830 (23.5)	6143 (91.9)	2819 (90.6)	3324 (92.9)	0.22
Keeping pets (yes)	2198 (28.3)	1850 (33.2)	872 (33.2)	978 (33.1)	0.66
ETS exposure at home (yes)	3542 (45.6)	662 (15.6)	313 (15.4)	349 (15.8)	0.99
Respiratory tract infections (yes)	2632 (33.9)	3230 (62.8)	1512 (62.8)	1718 (62.8)	0.84
Wheezing (yes)	2860 (36.8)	1482 (30.2)	691 (30.0)	791 (30.3)	0.83

Values are absolute numbers (percentages) for categorical variables. *Tested for differences in characteristics in intervention and control group using multinomial regression adjusted for randomisation stratum. Characteristics established using postal questionnaires during pregnancy included: *smoking during pregnancy* (yes, no), *maternal atopy* (yes, no), *maternal ethnicity* (Dutch, non-Western, other-Western) and *maternal educational level*. The Dutch Standard Classification of Education was used to categorise women’s self-reported highest education qualification [21]: low (less than 4 years of high school), middle (college), and high (Bachelor’s degree, Master’s degree). Data on net household income were available at the 2^nd^ year of life. *Birth weight* (grams) and *gestational age at birth* (weeks) were obtained from medical records. Postnatal factors were established using questionnaires and included: *breastfeeding ever* at age 0–6 months (yes, no); *keeping pets* (yes, no) at the 1^st^ year of life; *ETS exposure* at home (yes, no) measured at age 0–6 months; *respiratory tract infections* (yes, no) *and wheezing* (yes, no) at the 1^st^ year of life.

### Asthma (Related) Outcomes

At age 6 years, multilevel regression analysis indicated no differences in asthma, wheezing frequency, FeNO or Rint measurements between the intervention and control group (p>0.05) (Table 2 and 3).

**Table 2:** Intention-to-treat analyses: Prevalence and effect estimates of primary and secondary outcomes at age 6 years follow-up by allocation group.

	Intervention n = 3596	Care as usual n = 4179	Adjusted effect estimates [95% CI]*
*Primary outcome at age 6 years*			
Physician-diagnosed asthma ever^a^	86/2143 (4.0)	101/2425 (4.2)	1.01 (0.76–1.35)
*Secondary outcomes at age 6 years*			
Wheezing frequency^a^			
Never	1958/2143 (91.4)	2215/2425 (91.3)	Reference
1–3 times/year	143/2143 (6.7)	157/2425 (6.5)	1.02 (0.79,1.31)
>3 times/year	42/2143 (2.0)	53/2425 (2.2)	0.99 (0.71,1.37)
Health-related quality of life (CHQ-PF28 scales)^b^			
Physical functioning	97.30±11.16	97.22±11.17	0.00 (−0.01,0.01)
Role functioning: emotional/behaviour	97.40±10.78	97.59±10.28	0.00 (−0.01,0.00)
Role functioning: physical^d^	97.34±11.41	97.34±11.64	0.00 (−0.01,0.01)
Bodily pain	86.46±16.71	85.96±17.47	0.01 (−0.01,0.02)
General behaviour^d^	70.72±15.20	71.44±14.68	0.00 (−0.02,0.03)
Mental health^d^	81.65±14.53	81.90±14.43	0.00 (−0.02,0.02)
Self esteem^d^	83.81±15.31	83.35±15.28	0.01 (−0.01,0.03)
General health perceptions	87.19±15.82	86.78±15.74	0.00 (−0.02,0.02)
Parental impact: emotional	88.76±14.89	89.06±14.52	−0.01 (−0.02,0.01)
Parental impact: time	95.83±11.89	95.36±13.12	0.00 (−0.01,0.01)
Family activities	90.81±16.34	90.50±16.23	0.00 (−0.01,0.01)
Family cohesion	76.31±18.99	76.25±17.94	0.00 (−0.03,0.02)
Change in health^d^	56.15±15.46	56.84±16.28	−0.01 (−0.06,0.04)
Physical summary score^d^	57.36±6.22	57.19±6.29	0.17 (−0.58,0.93)
Psychosocial summary score^d^	53.03±6.79	53.08±6.66	−0.08 (0.53,0.37)
FeNO^c^^,^ d	7.20 (0.10–101.00)	7.30 (0.10–119.00)	−0.01 (−0.06,0.03)
Rint^c^^,^ d	0.93 (0.13–2.43)	0.93 (0.19–2.32)	0.09 (−0.17,0.35)
ETS exposure at home^a^	567/2840 (20.0)	745/3269 (22.8)	0.82 (0.66,1.03)

a Data are numerator/denominator (%).

b Mean ± standard deviation.

c Median (range).

d No baseline measurement available. Numbers of children does not equal the sum of the denominators in each subgroup because only those with baseline and follow-up data are included. Measurements on FeNO and Rint were available for respectively 3497 (45%) and 3828 (49%) of the participating children. FeNO = Fractional exhaled Nitric Oxide, Rint = airway resistance, ETS = Environmental Tobacco Smoke. *Adjusted for randomisation stratum, and baseline prevalence of outcomes. Care as usual is the reference group.

**Table 3:** Per-protocol analyses: Prevalence and effect estimates of primary and secondary outcomes at age 6 years follow-up by allocation group.

	Intervention n = 2718	Care as usual n = 3497	Adjusted effect estimates [95% CI]*
*Primary outcome at age 6 years*			
Physician-diagnosed asthma ever^a^	69/1704 (4.0)	87/1987 (4.4)	0.98 (0.72,1.34)
*Secondary outcomes at age 6 years*			
Wheezing frequency^a^			
Never	1565/1704 (91.8)	1808/1987 (91.0)	Reference
1–3 times/year	107/1704 (6.3)	134/1987 (6.7)	0.96 (0.73,1.28)
>3 times/year	32/1704 (1.9)	45/1987 (2.3)	0.96 (0.67,1.38)
Health-related quality of life (CHQ-PF28 scales)^b^			
Physical functioning	97.48±10.54	97.21±10.97	0.00 (−0.01,0.01)
Role functioning: emotional/behaviour	97.52±10.70	97.64±10.06	0.00 (−0.01,0.00)
Role functioning: physical^d^	97.52±10.99	97.20±12.03	0.00 (−0.01,0.01)
Bodily pain	86.46±16.78	85.75±17.62	0.01 (−0.01,0.02)
General behaviour^d^	70.89±15.22	71.61±14.66	0.00 (−0.02,0.03)
Mental health^d^	81.72±14.50	81.91±14.43	0.01 (−0.02,0.03)
Self esteem^d^	83.90±15.32	83.26±15.16	0.01 (−0.01,0.03)
General health perceptions	87.64±15.05	86.58±15.82	0.00 (−0.02,0.03)
Parental impact: emotional	89.07±14.70	89.00±14.60	0.00 (−0.02,0.02)
Parental impact: time	95.97±11.77	95.20±13.50	0.00 (−0.01,0.01)
Family activities	91.01±16.05	90.60±16.04	0.00 (−0.01,0.01)
Family cohesion	76.52±18.74	76.25±17.90	0.00 (−0.03,0.03)
Change in health^d^	56.06±15.20	57.10±16.45	−0.02 (−0.07,0.03)
Physical summary score^d^	57.49±5.87	57.11±6.34	0.36 (−0.37,1.10)
Psychosocial summary score^d^	53.08±6.78	53.09±6.61	−0.07 (0.63,0.50)
FeNO^c^^,^ d	7.30 (0.10–78.60)	7.40 (0.10–119.00)	−0.01 (−0.06,0.03)
Rint^c^^,^ d	0.93 (0.13–2.43)	0.93 (0.19–2.32)	−0.01 (−0.30,0.28)
ETS exposure at home^a^	417/2226 (18.7)	642/2704 (23.7)	**0.71 (0.59,0.87)** e

*Adjusted for randomisation stratum, and baseline prevalence of outcomes. Care as usual is the reference group.

a Data are numerator/denominator (%).

b Mean ± standard deviation.

c Median (range).

d No baseline measurement available.

e Applying Bonferroni correction: we performed 20 comparisons. At p = 0.0025 (i.e. 0.05/20), the decreased risk on ETS exposure at home ever in the intervention group remained statistically significant.

Numbers of children does not equal the sum of the denominators in each subgroup because only those with baseline and follow-up data are included. Measurements on FeNO and Rint were available for respectively 3497 (45%) and 3828 (49%) of the participating children. FeNO = Fractional exhaled Nitric Oxide, Rint = airway resistance, ETS = Environmental Tobacco Smoke.

### HRQOL

The response rate regarding the CHQ-PF28 scales at age 6 years was different for each scale and varied between 57–59% (n = 4410–4590). Baseline measurements were available for 8 out of 13 CHQ-PF28 scales. At age 6 years, no differences in HRQOL were found between the intervention and control group, after adjustment for baseline HRQOL and randomisation stratum (p>0.05) (Table 2 and 3).

### ETS Exposure: Baseline to Follow-up

**Figure 2:**
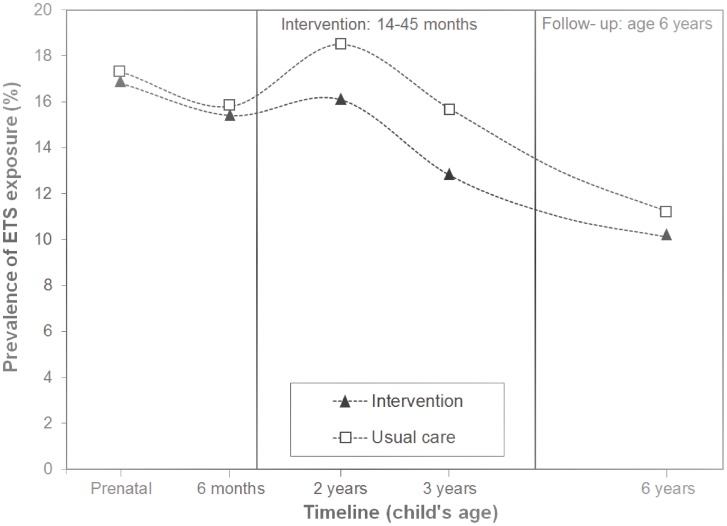
Prevalence of ETS exposure at home of intervention and control (usual care) group by child’s age (Intention-to-treat analysis). ETS exposure at home was defined based on the question ‘Do people smoke occasionally at home?’. Values are percentages and were tested for differences in characteristics in intervention and control group using logistic regression analyses. Population for analysis (N) and *P* values: Prenatal (N = 5598): p>0.05, 6 months (N = 4233): p>0.05, age 2 years (N = 5290): p = 0.02, age 3 years (N = 4894): p = 0.004, age 6 years (N = 4604): p>0.05.

Figure 2 shows the prevalence of ETS exposure at home before (fetal life to age 6 months), during (at age 14–45 months) and after (at age 6 years) the study period (according to the intention-to-treat analysis). During fetal life and at age 6 months, the prevalence of ETS exposure at home was around 16% in both the intervention and control group (p>0.05). At age 2 years, ETS exposure at home to children participating in the intervention group remained similar, but increased to 19% in the control group. At age 2, 3 and 6 years, the prevalence of ETS exposure at home was higher in children participating in the control group (age 2 years: p = 0.02, age 3 years: p = 0.004, age 6 years: p>0.05).

No differences in ETS exposure at home at age 2 and 3 years were found between intervention and control group after adjustment for baseline ETS exposure at home (reported during fetal life) using multinomial regression in an intention-to-treat analysis, (adjusted Odds Ratio [aOR] = 0.90, 95% Confidence Interval [CI]:0.74–1.08 at age 2 years and aOR = 0.81, 95% CI:0.66–1.01 at age 3 years). However, in the per-protocol analysis (n = 1560), multinomial regression analysis indicated a decreased risk on ETS exposure at home in the intervention group at age 2 and 3 years (aOR = 0.78, 95% CI:0.63–0.96 at age 2 years and aOR = 0.73, 95% CI:0.57–0.93 at age 3 years).

### ETS Exposure: Outcome

At age 6 years, no differences between intervention and control group were found on the outcome ‘ETS exposure at home ever’ using multilevel regression in an intention-to-treat analysis including adjustment for baseline ETS exposure at home (reported during fetal life) (aOR = 0.82, 95% CI:0.66–1.03) (Table 2). However, in an explorative per-protocol analysis, children who received the intervention at the intervention well-child centres had a decreased risk on ‘ETS exposure at home ever’ compared to children who visited the control well-child centres and who did not receive the intervention (aOR = 0.71, 95% CI:0.59–0.87) (Table 3).

### Interactions

No interaction effects on the outcomes were found of the research condition (intervention or control group) with socio-demographic characteristics or baseline values of the outcomes (p>0.10) (data not shown). We found no effect of the frequency of the intervention on outcomes.

### Process Evaluation of the Intervention

In total, professionals at well-child centres completed 6826 forms to assess asthma-like symptoms and ETS exposure for 2718 children (75.6% of the 3596 children) participating in the intervention group; and 1566 forms were completed for 682 children (16.3% of the 4179 children) participating in the control group (see discussion). In half of the children participating in the intervention group, the brief assessment form was applied at age 14 months (online repository Table S1). In total, the brief assessment form was never applied to 25% of the children participating in the intervention group. To 12% of the children participating in the intervention group, the brief assessment form was applied at each regularly scheduled visit up to year 4 (online repository Table S2).

Of the children in the intervention group who had ≥3 episodes of asthma-like symptoms in the past year, based on the data of the assessment forms, 53% (162/308) was already treated by general practitioner or paediatrician. Of the children with ≥3 episodes of asthma-like symptoms in the past year and asthma-like symptoms during the past month, 86% (119/139) was already treated by general practitioner or paediatrician.

Using the assessment forms, well-child professionals in the intervention group reported a decreasing prevalence of ETS exposure to children participating in the intervention group with increasing child’s age: 19% (276/1447) at the age of 14 months, 16% (266/1627) at age 24 months, 17% (301/1767) at age 36 months and 13% (225/1760) at age 45 months. At age 14 months, 89% (245/276) of the children with ETS exposure received the information leaflet regarding the prevention of ETS exposure. However, after the first year, the information leaflet regarding prevention of ETS exposure was less often provided to the parents of children who were exposed to ETS: 61% (163/266) at age 24 months, 64% (192/301) at age 36 months and 53% (119/225) at age 45 months.

## Discussion

Systematic assessment of asthma-like symptoms and ETS exposure by professionals at well-child centres, followed by counselling (when indicated - including referral to asthma nurse/general practitioner and providing parents with information leaflets on avoiding ETS exposure) did not lead to a lower prevalence of physician-diagnosed asthma ever, reduction in parent-reported wheezing symptoms and did not improve FeNO, Rint or parent-reported HRQOL at age 6 years. A decreased risk on ETS exposure at home in the intervention group was found at age 2 and 3 years, but at age 6 years no difference between intervention and control group was found. Process evaluation results showed that most children with wheezing were already treated by their general practitioner or by a paediatrician. Further, half of the parents of children with ETS exposure participating in the intervention group did not receive the information leaflets on ETS exposure at the intervention centres at age 45 months.

This is a community health worker-delivered intervention study using physician-diagnosed asthma ever, wheezing frequency, FeNO, Rint, HRQOL and (in addition) ETS exposure at home ever at age 6 years as outcomes. In contrast to the positive outcomes associated with community health worker-delivered interventions (including decreased asthma-like symptoms) reported by Postma et al [7], our study did not show a lower prevalence of asthma or wheezing after follow-up until age 6 years. Maybe more intensive counselling or interventions based on social cognitive theory, are required to achieve an effect on the asthma related outcomes. By using FeNO and Rint as outcomes we could evaluate the effect of the intervention on airway inflammation and lung function at age 6 years [37], [38], but no effect could be demonstrated. No differences in parent-reported HRQOL were found between intervention and control group, which possibly can be explained by the fact that the intervention did not reduce wheezing.

In addition to the review by Priest et al [39], showing that intensive and repeated counselling interventions seem to be promising to reduce ETS exposure, we found a transient effect of brief counselling aimed to avoid ETS exposure in children at preschool age. To increase efficiency of well-child visits, low intensive and brief assessments and health promotion interventions are preferred. However, process evaluation results showed that half of the parents of children with ETS exposure did not receive the information leaflet regarding prevention of ETS exposure at age 45 months. Apparently, for unknown reason, once prevention of ETS exposure was applied at the first year of life, professionals at well-child care did not tend to repeat the intervention later on while repeated feedback seems to be most effective to reduce the proportion of parents quitting smoking [40], [41].

The strengths of this study include the integration in current practice with a brief assessment form regarding asthma-like symptoms and ETS exposure, the large number of parents participating, the longitudinal design (with follow-up until child age 6 years) and large number of FeNO and Rint measurements. Limitations include shortcomings in the application of the brief assessment forms and counselling. Possible reasons are falling attendance of parents to the well-child centre; lack of time or priority is given to other health questions during the well-child visit or professionals who are not familiar with the intervention, that is still not routine practice. In this study, the professionals were provided with a two-hour specific training on how to apply and use the brief assessment form regarding asthma-like symptoms and ETS exposure. This level of instruction may not be optimal as we did not organize refreshment sessions nor provided feedback on performance or assessed its effect [42].

The study faced some difficulties. In contrast to what was described in our study protocol [12], data on inhaled steroids prescribed by a physician was not available at age 6 years. Asthma at age 6 years was defined as physician-diagnosed asthma ever, obtained by a parent reported questionnaire. In the future, at child’s age 10 years, data on inhaled steroids will be available and we recommend repeating the analyses at age 10 years.

In addition to the proposed outcomes, we evaluated whether the intervention had reduced ETS exposure at home. Children participating in the control group also visited the intervention well-child centres and systematic assessment and (when indicated) counselling of asthma-like symptoms and ETS exposure was applied to the parents of these children. Contamination of intervention and control condition may possibly have occurred by moving to another neighbourhood in the city and visiting another well-child centre. Because this contamination may have reduced the differences in results between intervention and control group, we amended the study protocol and in addition to the intention-to-treat analysis we performed a per-protocol analysis.

The following limitations would be a possible explanation for the negative study results: the study included a relatively low-intensity counselling intervention. However, the systematic assessment of the presence of asthma-like symptoms in early childhood by well-child professionals was prioritized and was considered feasible and essential in the Dutch youth healthcare system [43]. Another explanation for the negative study results is that there may have been a lack of intervention by the well-child care professional, and also by the parents/children (to only 12% of the children participating in the intervention group, the brief assessment form was applied at each regularly scheduled visit up to year 4 (Table S2)). Finally, since we used parent reports regarding the presence of asthma symptoms, HRQOL and ETS exposure at home, we may have lost precision.

We consider selection bias unlikely because a multiple imputed analysis including all eligible children did not change the results. Information bias should be considered for different measurements. Although the validity of assessing ETS exposure by questionnaires in epidemiological studies has been shown, misclassification may occur due to underreporting [44]. However, the use of biomarkers of tobacco smoke exposure in urine, saliva or blood, or nicotine in indoor air seems not superior to self-report [44]–[47]. We have to take into account the impact of parental symptom perception and, possibly, misclassification in their reports on asthma diagnosis and symptoms. Parental reports of wheezing are widely accepted in epidemiological studies and reliably reflects the incidence of wheezing in preschool children [14]. However, some misclassification cannot be excluded [48].

The decreased risk on ‘ETS exposure at home ever’ in the intervention group remained statistically significant even after correction for multiple testing.

This study raises questions about whether it is feasible to prevent the development of asthma by using systematic assessment and counselling of asthma-like symptoms and ETS exposure by using brief forms at well-child centres. We recommend further studies to evaluate whether professionals at well-child centres can contribute to optimal asthma management in other ways, and efforts are needed to optimize the protocols that can be implemented in this setting.

We also recommend further studies to improve the current intervention to optimise asthma management at well-child care. Based on previous results, it is recommended that professionals at well-child centres encourage breastfeeding and advise parents of children at high-risk of developing asthma to avoid ETS and indoor allergens exposure to their children to reduce the prevalence of asthma [3], [49]. To optimise asthma management and realise uniformity of practice at well-child care, future opportunities are the development of an assessment to estimate the risk of developing asthma at school age [50]. Further, we stress the importance to ban smoking in public places and residential settings to reduce children’s exposure to tobacco smoke.

Our study was embedded within the Dutch system of preventive health care provided by well-child centres in Rotterdam, the Netherlands. This may have consequences for the generalisability of our results in other areas and countries and therefore evaluation of our study in other, varied populations is recommended.

## Conclusion

A systematic assessment of asthma-like symptoms and ETS exposure by using brief assessment forms at well-child centres was not effective in reducing the prevalence of physician-diagnosed asthma ever and wheezing, and did not improve FeNO, Rint or HRQOL at age 6 years. Our results hold promise for interviewing parents and using information leaflets at well-child centres to reduce ETS exposure at home in preschool children.

## Supporting Information

Table S1 Age at enrolment in intervention group (N = 3596).
(DOCX)

Table S2 Frequency of applied intervention to preschool children participating in the intervention group (N = 3596).
(DOCX)

Checklist S1 CONSORT Checklist.
(DOCX)

File S1 Study design paper (BMC Public Health, 2010).
(PDF)

Protocol S1 Original Study Protocol.
(PDF)
