# Supplementary material for: Evaluation of Systematic Assessment of Asthma-Like Symptoms and Tobacco Smoke Exposure in Early Childhood by Well-Child Professionals: A Randomised Trial
Source: PLoS One. 2014 Mar 13;9(3):e90982. doi: 10.1371/journal.pone.0090982 (PMC3953324; doi:10.1371/journal.pone.0090982)
Supplement: Table S1 — Age at enrolment in intervention group (N = 3596). (DOCX) [file pone.0090982.s001.docx]

**SUPPORTING INFORMATION PONE-D-13-39597**

| **Table S1.** Age at enrollment in intervention group (N=3596). | |
| --- | --- |
| **Age at enrollment in intervention*** |  |
| 14 months | 1447 (53.4) |
| 24 months | 659 (24.3) |
| 36 months | 506 (18.7) |
| 45 months | 99 (3.7) |
| Values are absolute numbers (percentages). *Intervention = brief assessment form regarding asthma-like symptoms and environmental tobacco smoke exposure. Percentage of missing data on age at enrolment in the intervention group (N=3596): 24.6% (n=885). | |
