# Supplementary material for: Evaluation of Systematic Assessment of Asthma-Like Symptoms and Tobacco Smoke Exposure in Early Childhood by Well-Child Professionals: A Randomised Trial
Source: PLoS One. 2014 Mar 13;9(3):e90982. doi: 10.1371/journal.pone.0090982 (PMC3953324; doi:10.1371/journal.pone.0090982)
Supplement: Table S2 — Frequency of applied intervention to preschool children participating in the intervention group (N = 3596). (DOCX) [file pone.0090982.s002.docx]

**SUPPORTING INFORMATION PONE-D-13-39597**

| **Table S2.** Frequency of applied intervention to preschool children participating the intervention group (N=3596). | |
| --- | --- |
| **Frequency of applied intervention* during preschool age** |  |
| Never | 885 (24.6) |
| Once | 498 (13.8) |
| 2 times | 962 (26.8) |
| 3 times | 825 (22.9) |
| 4 times | 426 (11.8) |
| Values are absolute numbers (percentages). *Intervention = brief assessment form regarding asthma-like symptoms and environmental tobacco smoke exposure at age 14 or 24, 36, 45 months. | |
