# Supplementary material for: Evaluation of Systematic Assessment of Asthma-Like Symptoms and Tobacco Smoke Exposure in Early Childhood by Well-Child Professionals: A Randomised Trial
Source: PLoS One. 2014 Mar 13;9(3):e90982. doi: 10.1371/journal.pone.0090982 (PMC3953324; doi:10.1371/journal.pone.0090982)
Supplement: Checklist S1 — CONSORT Checklist. (DOCX) [file pone.0090982.s003.docx]

CONSORT 2010 checklist of information to include when reporting a randomised trial*


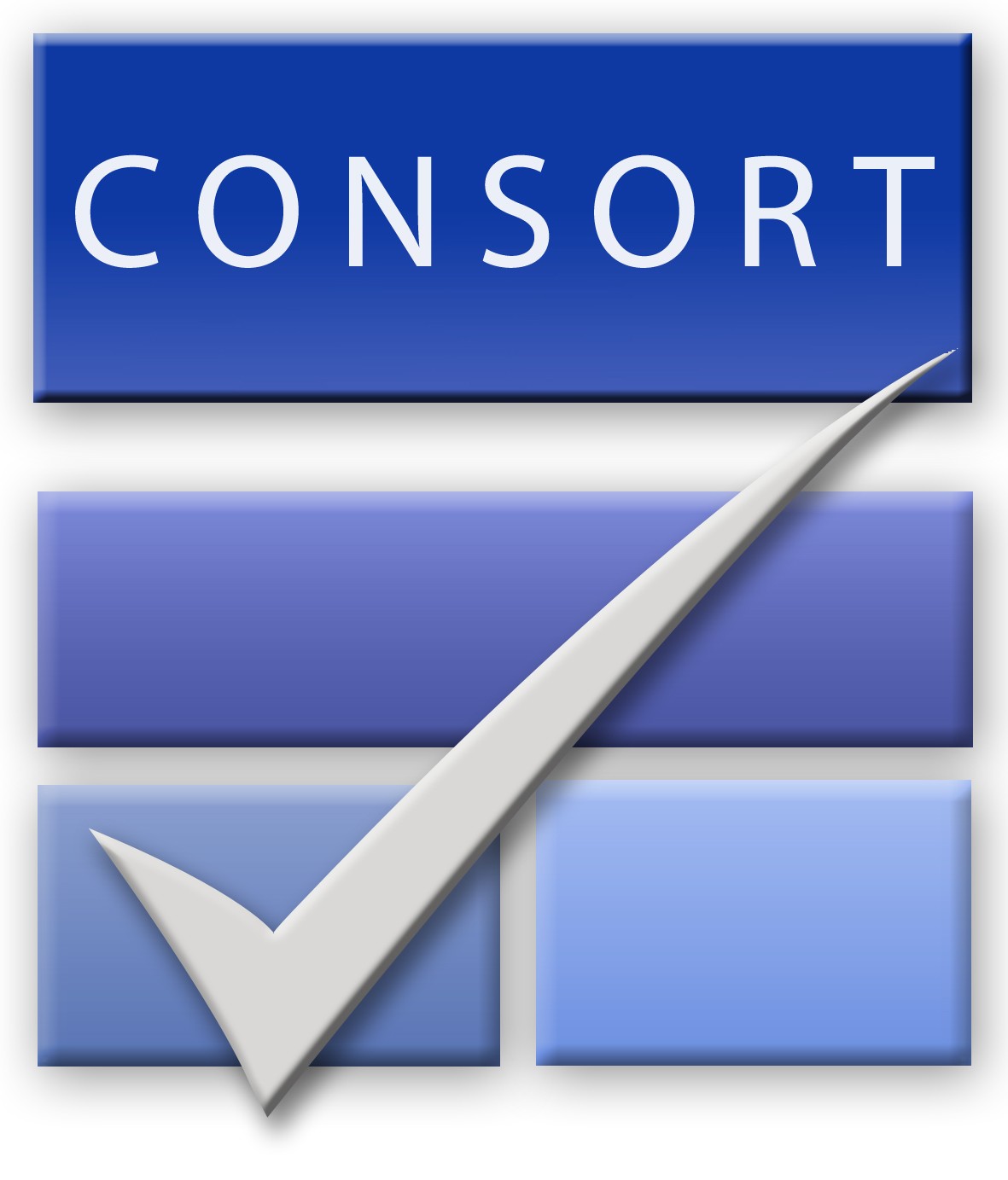


| Section/Topic | Item No | Checklist item | Reported on page No |
| --- | --- | --- | --- |
| Title and abstract | | | |
|  | 1a | Identification as a randomised trial in the title | see Title |
|  | 1b | Structured summary of trial design, methods, results, and conclusions (for specific guidance see CONSORT for abstracts) | see Abstract |
| Introduction | | | |
| Background and objectives | 2a | Scientific background and explanation of rationale | see Introduction, 1^st^ and 2^nd^ paragraph |
|  | 2b | Specific objectives or hypotheses | see Introduction, 3^rd^ paragraph |
| Methods | | | |
| Trial design | 3a | Description of trial design (such as parallel, factorial) including allocation ratio | see Methods section, 2^nd^ paragraph (‘Study design’) |
|  | 3b | Important changes to methods after trial commencement (such as eligibility criteria), with reasons | not applicable |
| Participants | 4a | Eligibility criteria for participants | see Methods section, 2^nd^ paragraph (‘Study design’) and Fig. 1 |
|  | 4b | Settings and locations where the data were collected | see Methods section, 2^nd^ paragraph (‘Study design’) |
| Interventions | 5 | The interventions for each group with sufficient details to allow replication, including how and when they were actually administered | see Methods section, paragraphs of subheading ‘Intervention and Usual care’. |
| Outcomes | 6a | Completely defined pre-specified primary and secondary outcome measures, including how and when they were assessed | see Methods section, subheading ‘Primary and secondary outcomes’. |
|  | 6b | Any changes to trial outcomes after the trial commenced, with reasons | see Methods section, subheading ‘Statistical analyses’, 2^nd^ paragraph. |
| Sample size | 7a | How sample size was determined | Sample size was reported in design paper |
|  | 7b | When applicable, explanation of any interim analyses and stopping guidelines | not applicable |
| Randomisation: |  |  |  |
| Sequence generation | 8a | Method used to generate the random allocation sequence | see Methods section, 2^nd^ paragraph (‘Study design’) |
|  | 8b | Type of randomisation; details of any restriction (such as blocking and block size) | Not applicable |
| Allocation concealment mechanism | 9 | Mechanism used to implement the random allocation sequence (such as sequentially numbered containers), describing any steps taken to conceal the sequence until interventions were assigned | see Methods section, 2^nd^ paragraph (‘Study design’) |
| Implementation | 10 | Who generated the random allocation sequence, who enrolled participants, and who assigned participants to interventions | see Methods section, 2^nd^ paragraph (‘Study design’) |
| Blinding | 11a | If done, who was blinded after assignment to interventions (for example, participants, care providers, those assessing outcomes) and how | see Methods section, 2^nd^ paragraph (‘Study design’) |
|  | 11b | If relevant, description of the similarity of interventions | Not applicable |
| Statistical methods | 12a | Statistical methods used to compare groups for primary and secondary outcomes | see Methods section, subheading ‘Statistical analyses’. |
|  | 12b | Methods for additional analyses, such as subgroup analyses and adjusted analyses | see Methods section, subheading ‘Statistical analyses’. |
| Results | | | |
| Participant flow (a diagram is strongly recommended) | 13a | For each group, the numbers of participants who were randomly assigned, received intended treatment, and were analysed for the primary outcome | see Results section, subheading ‘Recruitment’and Fig. 1 |
|  | 13b | For each group, losses and exclusions after randomisation, together with reasons | Fig 1. |
| Recruitment | 14a | Dates defining the periods of recruitment and follow-up | see Methods section, 2^nd^ paragraph (‘Study design’) |
|  | 14b | Why the trial ended or was stopped | see Methods section, 2^nd^ paragraph (‘Study design’) |
| Baseline data | 15 | A table showing baseline demographic and clinical characteristics for each group | Table 1 |
| Numbers analysed | 16 | For each group, number of participants (denominator) included in each analysis and whether the analysis was by original assigned groups | Table 2 |
| Outcomes and estimation | 17a | For each primary and secondary outcome, results for each group, and the estimated effect size and its precision (such as 95% confidence interval) | Table 2 |
|  | 17b | For binary outcomes, presentation of both absolute and relative effect sizes is recommended | Table 2 |
| Ancillary analyses | 18 | Results of any other analyses performed, including subgroup analyses and adjusted analyses, distinguishing pre-specified from exploratory | Table 3 |
| Harms | 19 | All important harms or unintended effects in each group (for specific guidance see CONSORT for harms) | Not applicable |
| Discussion | | | |
| Limitations | 20 | Trial limitations, addressing sources of potential bias, imprecision, and, if relevant, multiplicity of analyses | see Discussion section, 5^th^-9^th^ paragraph |
| Generalisability | 21 | Generalisability (external validity, applicability) of the trial findings | see last paragraph of the Discussion section |
| Interpretation | 22 | Interpretation consistent with results, balancing benefits and harms, and considering other relevant evidence | see Discussion section, 2^nd^ and 3th paragraph |
| Other information | | |  |
| Registration | 23 | Registration number and name of trial registry | see end of Abstract |
| Protocol | 24 | Where the full trial protocol can be accessed, if available | see Methods section, the end of 2^nd^ paragraph (‘Study design’) |
| Funding | 25 | Sources of funding and other support (such as supply of drugs), role of funders | see ‘Financial Disclosure’ |

*We strongly recommend reading this statement in conjunction with the CONSORT 2010 Explanation and Elaboration for important clarifications on all the items. If relevant, we also recommend reading CONSORT extensions for cluster randomised trials, non-inferiority and equivalence trials, non-pharmacological treatments, herbal interventions, and pragmatic trials. Additional extensions are forthcoming: for those and for up to date references relevant to this checklist, see [www.consort-statement.org](http://www.consort-statement.org).
