# Supplementary material for: Evaluation of Systematic Assessment of Asthma-Like Symptoms and Tobacco Smoke Exposure in Early Childhood by Well-Child Professionals: A Randomised Trial
Source: PLoS One. 2014 Mar 13;9(3):e90982. doi: 10.1371/journal.pone.0090982 (PMC3953324; doi:10.1371/journal.pone.0090982)
Supplement: File S1 — Study design paper (BMC Public Health, 2010). (PDF) [file pone.0090982.s004.pdf]

Hafkamp-de Groen et al. BMC Public Health 2010, 10:555
http://www.biomedcentral.com/1471-2458/10/555

BMC Public Health

STUDY PROTOCOL Open Access

Early detection and counselling intervention of asthma symptoms in preschool children: study design of a cluster randomised controlled trial

Esther Hafkamp-de Groen[1,2*], Ashna D Mohangoo[1,2,3], Johan C de Jongste[4], Johannes C van der Wouden[5], Henriëtte A Moll[4], Vincent WV Jaddoe[1,4,6], Albert Hofman[6], Harry J de Koning[2], Hein Raat[2]

Abstract

Background: Prevention of childhood asthma is an important public health objective. This study evaluates the effectiveness of early detection of preschool children with asthma symptoms, followed by a counselling intervention at preventive child health centres. Early detection and counselling is expected to reduce the prevalence of asthma symptoms and improve health-related quality of life at age 6 years.

Methods/design: This cluster randomised controlled trial was embedded within the Rotterdam population-based prospective cohort study Generation R in which 7893 children (born between April 2002 and January 2006) participated in the postnatal phase. Sixteen child health centres are involved, randomised into 8 intervention and 8 control centres. Since June 2005, an early detection tool has been applied at age 14, 24, 36 and 45 months at the intervention centres. Children who met the intervention criteria received counselling intervention (personal advice to parents to prevent smoke exposure of the child, and/or referral to the general practitioner or asthma nurse). The primary outcome was asthma diagnosis at age 6 years. Secondary outcomes included frequency and severity of asthma symptoms, health-related quality of life, fractional exhaled nitric oxide and airway resistance at age 6 years. Analysis was according to the intention-to-treat principle. Data collection will be completed end 2011.

Discussion: This study among preschool children provides insight into the effectiveness of early detection of asthma symptoms followed by a counselling intervention at preventive child health centres.

Trial registration: Current Controlled Trials ISRCTN15790308.

Background

Asthma (symptoms)

Asthma is a highly prevalent chronic condition associated with considerable morbidity, reduced health-related quality of life (HrQoL) and significant costs for public health [1-6]. The World Health Organisation (WHO) defines asthma as a chronic inflammatory disorder of the airways associated with increased bronchial hyperresponsiveness [1]. The WHO recently estimated that worldwide about 300 million people suffer from asthma [1]. The International Study of Asthma and Allergies in Childhood (ISAAC) showed marked variations in the prevalence of childhood asthma between countries [5]. On average, 10% of European children suffer from asthma [1].

In preschool children it is difficult to diagnose asthma because symptoms are non-specific and additional tests are not yet possible. Therefore, a symptom-based rather than a diagnosis-based approach has been applied [7]. In preschool children asthma symptoms are commonly defined as wheezing, shortness of breath or dyspnea [8-10]. An asthma diagnosis is often preceded by asthma symptoms in the first years of life. In the Netherlands, the Prevention and Incidence of Asthma and Mite Allergy (PIAMA) study reported a wheezing prevalence

* Correspondence: e.hafkamp@erasmusmc.nl
[1]The Generation R Study Group, Erasmus MC, Rotterdam, The Netherlands
Full list of author information is available at the end of the article

© 2010 Hafkamp-de Groen et al; licensee BioMed Central Ltd. This is an Open Access article distributed under the terms of the Creative Commons Attribution License (http://creativecommons.org/licenses/by/2.0), which permits unrestricted use, distribution, and reproduction in any medium, provided the original work is properly cited.

of 21% in the children's first year, rapidly falling to 4% in the 4-5th years of age [11].

Child Health Care

Asthma symptoms are regularly underreported, and children often remain undiagnosed and/or undertreated [12-15]. The Netherlands has a unique preventive child health care system, i.e. about 90% of all children (aged 0-4 years) are periodically monitored in a nationwide programme at set ages [16]. This programme is offered free-of-charge by the government and participation is voluntary [17]. However, until now, no systematic early detection and counselling intervention of asthma symptoms has been applied in preventive child health care.

Objectives

This study evaluates the effectiveness of early detection of asthma symptoms in preschool children in preventive child health centres. Our hypothesis is that early detection of asthma symptoms (at ages 14, 24, 36 and 45 months) followed by a counselling intervention at the child health centre, will reduce the prevalence and severity of asthma symptoms and asthma, and also improve HrQoL at age 6 years [18-21].

Methods/design

Design and setting

This cluster randomised controlled trial (RCT) is embedded in the Generation R study, in collaboration with the regional Child Health Care Organisation *Ouder & Kindzorg* in Rotterdam. The Generation R study is a prospective population-based cohort study running from fetal life until young adulthood. The Generation R study is designed to identify early environmental and biological determinants of growth, development and health in fetal life and childhood; study details have been published [22-25].

The present study was conducted in accordance with the guidelines proposed in the Declaration of Helsinki, and is approved by the Medical Ethical Committee of the Erasmus Medical Centre. Written consent was obtained from all participating parents.

Participants

The Generation R cohort included 9778 pregnant women living in Rotterdam, the Netherlands. The participating women gave birth to 9745 live-born children between April 2002 and January 2006. A total of 7893 children participated in the postnatal phase [25]. The cohort for the early detection and counselling intervention of asthma symptoms consisted of all 7775 children participating in the postnatal phase of the Generation R study and living in the intervention area (Rotterdam-North, defined by postal codes 3010-3070) (Figure 1).

Randomisation

Randomisation was done at the level of the child health centres. First, the child health centres were ranked based on the socioeconomic status of their neighbourhood. Child health centers in each subsequent couple in this list were randomly assigned to the intervention group (n = 8) or the control group (n = 8) (Figure 1).

Intervention Condition

A - Early detection

At the intervention centres the physician (for children aged 14, 36 and 45 months) or the nurse (for children aged 24 months) performs the early detection tool in an interview with the parents during the regular visits. On average, the interview takes about 1 minute. There are 6 questions: 4 adapted from the ISAAC on the presence of asthma symptoms during the past 4 weeks and the past 12 months [26,27], and 2 on the use of anti-asthma therapy during the past 4 weeks prescribed by the general practitioner (GP) or paediatrician, and on tobacco smoke exposure [26]. Details on this early detection tool are given in Additional file 1.

B - Counselling intervention: Personal advice

When parents reported that their child had at least 3 episodes of asthma symptoms during the past 12 months and at least 1 episode of asthma symptoms in the past 4 weeks, they received an information leaflet concerning asthma. If the child had been free of asthma symptoms during the past 4 weeks, the physician advises a visit to the GP should the child's asthma symptoms return. If the child had been exposed to tobacco smoke, the physician/nurse advises parents to prevent this, and provides them with an information leaflet about preventing their child from exposure to smoke. Physicians/nurses at the child health centres use environmental (anti-asthma home) intervention guidelines for children already diagnosed with an allergy (Guidelines of the Dutch College of General Practitioners) [10] (see Additional file 2).

C - Counselling intervention: Referral

When parents reported that their child had at least 3 episodes of asthma symptoms during the past 12 months, of which at least 1 in the past 4 weeks, and the child has not yet been treated by the GP or paediatrician in the past 4 weeks, the child is immediately referred to the asthma nurse at the regional Health Care Organisation and the GP. If the child has already been treated by the GP or paediatrician in the past 4 weeks, the child is referred to the asthma nurse only (Additional file 2).

Control condition

The 'control' child health centres followed current routine practice. Although parents might spontaneously

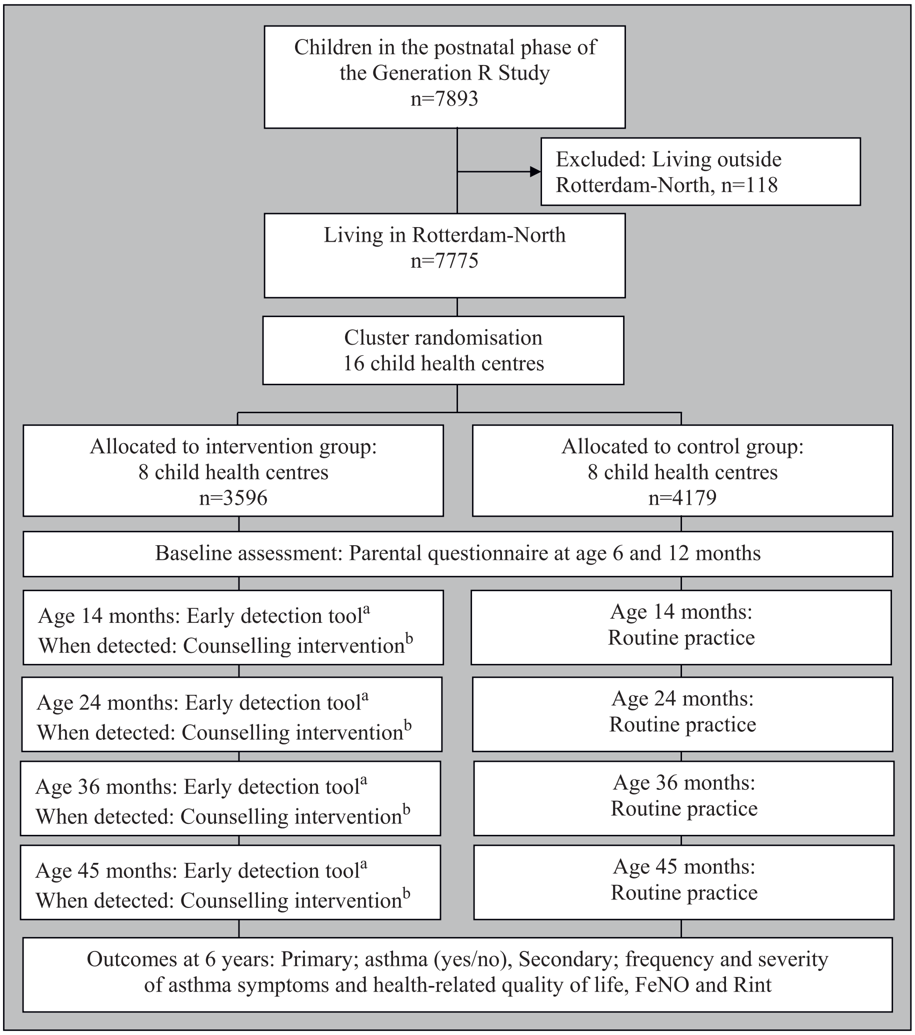

[a] See Additional file 1, [b] See Addditional file 2

Figure 1 Study design. The flow of participants through the randomised controlled trial for early detection of asthma symptoms and application of the counselling intervention.

mention asthma symptoms, or the physician/nurse might notice asthma symptoms, no active effort was made by the study team to facilitate detection of asthma symptoms in the control centres.

Measurements

Baseline assessment

Information on asthma symptoms was obtained via questionnaires at age 6 and 12 months, and yearly thereafter. Questionnaires were completed by the parents until the age of 6 years. Wheezing and breathlessness were measured with items adapted from the ISAAC [28,29]; the question on persistent phlegm ("having had phlegm on at least 4 days per week for at least 3 months") was based on the American Thoracic Society questionnaire for respiratory symptoms in childhood [29]. Information on parental smoking at baseline was obtained via a questionnaire during pregnancy, before randomisation.

Primary outcome

Both the intervention and control group are followed, and outcomes at age 6 years are compared to evaluate the effectiveness of early detection and counselling intervention of asthma symptoms. At age 6 years it is still difficult to diagnose asthma due to the absence of a gold standard. However, in many children with transient wheezing conditions other than asthma, the symptoms will have disappeared by this age; moreover, an asthma diagnosis is more accurate at age 6 years than in preschool children.

The following items (obtained via questionnaires) are used for the case definition of asthma: 1) at least 1 reported episode of wheezing, 2) inhaled steroids prescribed by a physician, 3) a parental report of a physician's diagnosis of asthma at any time plus a parental report of asthma during the past 12 months.

In the analyses, children are considered positive for asthma only if they have one or more positive items at the ages of 45 months and 6 years [30].

Secondary outcomes

Supplementary to this dichotomous primary outcome (asthma yes or no) we use continuous outcomes at age 6 years: i.e. frequency and severity of asthma symptoms, and HrQoL variables, obtained via questionnaires. To assess the overall impact of early detection and counselling intervention of children with asthma symptoms on HrQoL, the 28-item child health questionnaire 'parent form' (CHQ-PF28) is used at age 6 years [31].

At age 6 years, children are tested with i) measurement of fractional exhaled nitric oxide (FeNO), a marker of eosinophilic airway inflammation which is elevated in atopic asthma, and ii) Rint, a lung function test that measures interrupter resistance of the respiratory system [32]. Other outcomes obtained via questionnaires at age 12, 24, 36 and 48 months include HrQoL (the Infant-Toddler Quality of Life Questionnaire, ITQOL) at age 12, 24 and 48 months [33,34], and the Health Utilities Index Mark 3 (HUI3) at age 36 months [35-37].

Co-variates

Information on parental characteristics (age, ethnicity, educational level, household income, allergy, and presence of other conditions or diseases) are obtained from the first questionnaire at enrollment in the study. Parental smoking habits are assessed via questionnaires when the child is aged 6, 24 and 36 months, and 6 years. Child's birth weight, date of birth, gestational age and gender are obtained from national midwife and obstetrician registries. Breastfeeding and presence of pets are assessed by questionnaire at age 6 months. Other child characteristics (age, presence of siblings, day-care attendance, eczema, allergy, respiratory and non-respiratory tract infections, presence of other conditions or diseases, frequency and severity of asthma symptoms, and prevalence of physician-diagnosed asthma) are obtained via questionnaires at the age of 12, 24, 36 and 48 months, and 6 years.

Power of the study

Net 7775 children will visit the 16 participating child health centers. Considering a visit response of 90% [16] and assuming a loss-to-follow up of 30%, at least 2450 children per group will participate in outcome measurement at 6 years. Taking into account cluster randomization, assuming a prevalence of asthma of 12% in the control group at age 6 years [38,39], alpha 0.05 and a power of 0.80, an absolute difference in the prevalence of children with asthma between intervention and control group of 2.25% (12% asthma diagnosis in the intervention group, 9.75% asthma in the control group) can be established with a total of 16 child health centers/7775 children starting in the study at age 14 months.

Statistical analyses

The effectiveness of the early detection tool for asthma symptoms is evaluated on an intention-to-treat principle [40]. Multi-level analyses are applied to allow for dependency between the individual measurements within the 16 randomised child health centres [41,42]. Outcomes (primary and secondary) are analysed by means of logistic regression analysis with independent variables: intervention or control group, gender, age, socioeconomic status, ethnicity, exposure to tobacco smoke, pets, siblings, co-morbidity (e.g. eczema, allergy, respiratory and non-respiratory tract infections). Interaction effects of gender, social disadvantage and ethnic background are examined. Complementary subgroup analyses are done for gender, socioeconomic status and ethnicity. The impact of early detection and counselling intervention of asthma symptoms, as compared with the control group, is analyzed by means of multiple linear or logistic regression analysis, for continuous or dichotomous outcome variables, respectively [42]. A non-response analysis is conducted to determine possible selection bias. In the non-response analysis the following characteristics of (non)-participating children and their parents are taken into account: gender, ethnicity, socioeconomic status, frequency of asthma symptoms, exposure to tobacco smoke, use of asthma therapy, and abnormal lung auscultation. The trial is reported according to the CONSORT standards for reporting RCTs [41]. Statistical analyses are performed using the Statistical Package of Social Sciences version 17.0 for Windows (SPSS Inc, Chicago, IL, USA).

Discussion

We present the design of a cluster RCT for early detection of asthma symptoms in preschool children, followed by a counselling intervention at preventive child health centres. Although asthma often starts in early childhood [43,44], in most preschool children asthma can not reliably be diagnosed [18,45]. On the other hand, many young children do have asthma symptoms, and asthma may be underdiagnosed and/or undertreated in this group [14,46]. Diagnosing asthma is difficult in preschool children due to the nonspecific symptoms and because conventional lung function tests cannot be carried out [7].

Until now, there is no evidence that early detection and counselling interventions at young age alter the natural course of asthma [44]. However, it is known that impaired lung function is related to the length of the asthma disease process [47]. So far, evidence suggests that intervention during the early stages of asthma is important [47].

This study aimed to evaluate an early detection tool that is based on symptoms, and followed by a counselling intervention. The goal is to apply an early detection and intervention programme in child health centers to promote timely detection of asthma symptoms in preschool children, and thereby improve their wellbeing and HrQoL.

The ISAAC core questions were originally designed for epidemiological studies in children aged 6 years and over, and not for individual case-finding purposes. However, we used selected questions on the frequency of asthma symptoms, adapted from the ISAAC core questionnaires as they were originally used in the Dutch PIAMA cohort [48]. It remains debatable whether or not parents' reports on asthma symptoms are accurate [49-51]. Some state that asthma symptoms are reported with low or moderate accuracy [52,53], whereas others found that, compared with paediatricians' records, parents were able to report asthma symptoms accurately, especially for young children [54]. We decided to use early detection of the child's asthma symptoms by means of parental reporting, obtained via an interview conducted by the physician or nurse. As an early detection tool, parent-reported questionnaires are non-invasive, inexpensive and reliable. However, the impact of this programme remains to be shown and can only be accomplished based on a RCT, such as the present study.

The strengths of the present study are the size of the study population, the randomised controlled design conducted in the practice setting (which will facilitate implementation if the programme proves effective), information on numerous potential mediating factors/confounders, and the regular free-of-charge visits [16]. Children visit the child health centres at set ages, which offers optimal opportunity to provide tailored asthma symptom counselling.

Although lung function can be applied, and symptoms become more specific at age 6 years, it remains difficult to diagnose asthma at school age. The definition of asthma remains arbitrary and mainly symptom based. However, an asthma diagnosis is more evident at age 6 years compared to preschool age. Therefore, the primary end-point in this study is asthma (yes or no) at age 6 years, defined as parent-reported asthma symptoms, medication, or both at different ages, because the aim was to detect persistent asthma symptoms with clinical relevance, as defined by Caudri et al. [30]. Additionally, FeNO and Rint measurements are used as secondary outcomes. Both techniques have been well standardised for use in children older than 4 years by the American Thoracic Society and the European Respiratory Society [55,56].

In the Netherlands, the Child Health Care physicians and nurses play a central role in the early detection and counselling intervention of asthma symptoms in preschool children because they have routine contact with about 90% of all preschool children and their families [16]. In a well-regulated setting, administering a systematic early detection tool consisting of parents' reports of the child's asthma symptoms (elicited via an interview by the physician or nurse) may be an effective way of selecting children who might benefit from asthma counselling, more detailed assessment at the child health centre, or referral to an asthma nurse, GP or paediatrician.

Additional material

Additional file 1: Early detection tool for early detection of asthma symptoms in preschool children. The file contains the early detection tool (consisting of 6 questions).

Additional file 2: Counselling intervention scheme following early detection of asthma symptoms (I) and tobacco smoke exposure (II). The file contains an overview of the steps of counselling intervention, following early detection of asthma symptoms (I) and tobacco smoke exposure (II) in preschool children.

Acknowledgements

This study is funded by the Netherlands Organisation for Health Research and Development (ZonMw: project no. 22000128). The Generation R Study is conducted by the Erasmus MC, University Medical Center Rotterdam, in close collaboration with the Child Health Care *Ouder & Kindzorg* Rotterdam, the Rotterdam Homecare Foundation and the *Stichting Trombosedienst & Artsenlaboratorium* Rijnmond (STAR) Rotterdam. We gratefully acknowledge the contribution of all Generation R participants and the health care workers at the participating child health centres in Rotterdam, especially Inge Moorman, MD.

Author details

[1]The Generation R Study Group, Erasmus MC, Rotterdam, The Netherlands. [2]Department of Public Health, Erasmus MC, Rotterdam, The Netherlands. [3]TNO Quality of Life, Netherlands Organisation for Applied Scientific Research, Department Prevention and Care, Leiden, The Netherlands. [4]Department of Paediatrics, Erasmus MC-Sophia Children's Hospital, Rotterdam, The Netherlands. [5]Department of General Practice, Erasmus MC, Rotterdam, The Netherlands. [6]Department of Epidemiology, Erasmus MC, Rotterdam, The Netherlands.

Authors' contributions

The study was initiated by HR and HJK, and they were responsible for acquiring the study grant. ADM, JCJ, JCW, HJK and HR participated in the study concept and design. ADM set up the cluster randomised controlled trial and was responsible for data collection, data analysis and reporting the study results until June 2008. After June 2008 EH had full access to all the data in the study and takes responsibility for the integrity of the data and the accuracy of the data analysis and reporting the study results. All authors reviewed and approved the final version of this manuscript.

Competing interests

The authors declare that they have no competing interests.

Received: 6 September 2010 Accepted: 15 September 2010
Published: 15 September 2010

References

1. World Health Organization: **Bronchial asthma.** *World Health Organization Fact Sheet N° 307. Geneva* 2008.
2. Masoli M, Fabian D, Holt S, Beasley R, Global Initiative for Asthma (GINA) Program: **The global burden of asthma: executive summary of the GINA Dissemination Committee report.** *Allergy* 2004, **59(5)**:469-478.
3. Halterman JS, Yoos HL, Conn KM, Callahan PM, Montes G, Neely TL, Szilagyi PG: **The impact of childhood asthma on parental quality of life.** *J Asthma* 2004, **41(6)**:645-653.
4. Eder W, Ege MJ, von Mutius E: **The asthma epidemic.** *N Engl J Med* 2006, **355(21)**:2226-2235.
5. ISAAC Steering Committee: **Worldwide variations in the prevalence of asthma symptoms: the International Study of Asthma and Allergies in Childhood (ISAAC).** *Eur Respir J* 1998, **12(2)**:315-335.
6. Braman SS: **The global burden of asthma.** *Chest* 2006, **130(Suppl 1)**:4S-12S.
7. Bush A: **Diagnosis of asthma in children under five.** *Prim Care Respir J* 2007, **16(1)**:7-15.
8. Koopman LP, Brunekreef B, de Jongste JC, Neijens HJ: **Definition of respiratory symptoms and disease in early childhood in large prospective birth cohort studies that predict the development of asthma.** *Pediatr Allergy Immunol* 2001, **12(3)**:118-124.
9. Sistek D, Tschopp JM, Schindler C, Brutsche M, Ackermann-Liebrich U, Perruchoud AP, Leuenberger P: **Clinical diagnosis of current asthma: predictive value of respiratory symptoms in the SAPALDIA study. Swiss Study on Air Pollution and Lung Diseases in Adults.** *Eur Respir J* 2001, **17(2)**:214-219.
10. Bindels PJE, Van der Wouden JC, Ponsioen BP, Brand PLP, Salomé PL, Van Hensbergen W, Van Hasselt PA, Steenkamer TA, Grol MH: **Guidelines of the Dutch College of General Practitioners: asthma in children.** *Huisarts Wet* 2006, **49(11)**:557-572.
11. Wijga AH, Smit HA: **Astma bij peuters en kleuters: Resultaten van het PIAMA onderzoek.** *RIVM Report. Bilthoven* 2004.
12. Maziak W, von Mutius E, Beimfohr C, Hirsch T, Leupold W, Keil U, Weiland SK: **The management of childhood asthma in the community.** *Eur Respir J* 2002, **20(6)**:1476-1482.
13. Halterman JS, Aligne CA, Auinger P, McBride JT, Szilagyi PG: **Inadequate therapy for asthma among children in the United States.** *Pediatrics* 2000, **105(1 Pt 3)**:272-276.
14. Joseph CL, Foxman B, Leickly FE, Peterson E, Ownby D: **Prevalence of possible undiagnosed asthma and associated morbidity among urban schoolchildren.** *J Pediatr* 1996, **129(5)**:735-742.
15. Amado MC, Portnoy JM: **Recent advances in asthma management.** *Mo Med* 2006, **103(1)**:60-64.
16. Burgmeijer RJF, Van Geenhuizen YM, Filedt Kok-Weimar T, De Jager AM: **Op weg naar volwassenheid. Evaluatie Jeugdgezondheidszorg 1996.** *TNO/KPMG Report. Leiden/Maarssen* 1997.
17. Ministry of Health Welfare and Sport: **Basistakenpakket Jeugdgezondheidszorg 0-19 jaar.** *Report. Den Haag* 2003.
18. Amado MC, Portnoy JM: **Diagnosing asthma in young children.** *Curr Opin Allergy Clin Immunol* 2006, **6(2)**:101-105.
19. Jones A: **Screening for asthma in children.** *Br J Gen Pract* 1994, **44(381)**:179-183.
20. Lau S: **Transition from childhood to adult asthma.** *Lancet* 2008, **372(9643)**:1014-1015.
21. Lewis TC, Robins TG, Joseph CL, Parker EA, Israel BA, Rowe Z, Edgren KK, Salinas MA, Martinez ME, Brown RW: **Identification of gaps in the diagnosis and treatment of childhood asthma using a community-based participatory research approach.** *J Urban Health* 2004, **81(3)**:472-488.
22. Hofman A, Jaddoe VW, Mackenbach JP, Moll HA, Snijders RF, Steegers EA, Verhulst FC, Witteman JC, Büller HA: **Growth, development and health from early fetal life until young adulthood: the Generation R Study.** *Paediatr Perinat Epidemiol* 2004, **18(1)**:61-72.
23. Jaddoe VW, Bakker R, van Duijn CM, van der Heijden AJ, Lindemans J, Mackenbach JP, Moll HA, Steegers EA, Tiemeier H, Uitterlinden AG, Verhulst FC, Hofman A: **The Generation R Study Biobank: a resource for epidemiological studies in children and their parents.** *Eur J Epidemiol* 2007, **22(12)**:917-923.
24. Jaddoe VW, Mackenbach JP, Moll HA, Steegers EA, Tiemeier H, Verhulst FC, Witteman JC, Hofman A: **The Generation R Study: Design and cohort profile.** *Eur J Epidemiol* 2006, **21(6)**:475-484.
25. Jaddoe VW, van Duijn CM, van der Heijden AJ, Mackenbach JP, Moll HA, Steegers EA, Tiemeier H, Uitterlinden AG, Verhulst FC, Hofman A: **The Generation R Study: design and cohort update until the age of 4 years.** *Eur J Epidemiol* 2008, **23(12)**:801-811.
26. Asher MI, Keil U, Anderson HR, Beasley R, Crane J, Martinez F, Mitchell EA, Pearce N, Sibbald B, Stewart AW, Strachan D, Weiland SK, Williams HC: **International Study of Asthma and Allergies in Childhood (ISAAC): rationale and methods.** *Eur Respir J* 1995, **8(3)**:483-491.
27. Asher MI, Weiland SK: **The International Study of Asthma and Allergies in Childhood (ISAAC). ISAAC Steering Committee.** *Clin Exp Allergy* 1998, **28(Suppl 5)**:52-66.
28. Solé D, Vanna AT, Yamada E, Rizzo MC, Naspitz CK: **International Study of Asthma and Allergies in Childhood (ISAAC) written questionnaire: validation of the asthma component among Brazilian children.** *J Investig Allergol Clin Immunol* 1998, **8(6)**:376-382.
29. Ferris BG: **Epidemiology Standardization Project (American Thoracic Society).** *Am Rev Respir Dis* 1978, **118(6 Pt 2)**:1-120.
30. Caudri D, Wijga A, A Schipper CM, Hoekstra M, Postma DS, Koppelman GH, Brunekreef B, Smit HA, de Jongste JC: **Predicting the long-term prognosis of children with symptoms suggestive of asthma at preschool age.** *J Allergy Clin Immunol* 2009, **124(5)**, 903-910e1-7.
31. Raat H, Botterweck AM, Landgraf JM, Hoogeveen WC, Essink-Bot ML: **Reliability and validity of the short form of the child health questionnaire for parents (CHQ-PF28) in large random school based and general population samples.** *J Epidemiol Community Health* 2005, **59(1)**:75-82.
32. Van Mastrigt E, Gabriele C, De Jongste JC: **Exhaled nitric oxide in infants–what is a nice test like FENO doing in a place like this?** *Semin Respir Crit Care Med* 2007, **28(3)**:264-271.
33. Raat H, Landgraf JM, Oostenbrink R, Moll HA, Essink-Bot ML: **Reliability and validity of the Infant and Toddler Quality of Life Questionnaire (ITQOL) in a general population and respiratory disease sample.** *Qual Life Res* 2007, **16(3)**:445-460.
34. Guillemin F, Bombardier C, Beaton D: **Cross-cultural adaptation of health-related quality of life measures: literature review and proposed guidelines.** *J Clin Epidemiol* 1993, **46(12)**:1417-1432.
35. Klassen AF, Landgraf JM, Lee SK, Barer M, Raina P, Chan HW, Matthew D, Brabyn D: **Health-related quality of life in 3 and 4 year old children and their parents: preliminary findings about a new questionnaire.** *Health Qual Life Outcomes* 2003, **1**:81.
36. Raat H, Bonsel GJ, Hoogeveen WC, Essink-Bot ML, Dutch HUI Group: **Feasibility and reliability of a mailed questionnaire to obtain visual analogue scale valuations for health states defined by the Health Utilities Index Mark 3.** *Med Care* 2004, **42(1)**:13-18.
37. Ware JE Jr, Sherbourne CD: **The MOS 36-item short-form health survey (SF-36). I. Conceptual framework and item selection.** *Med Care* 1992, **30(6)**:473-483.
38. Caudri D, Wijga A, Gehring U, Smit HA, Brunekreef B, Kerkhof M, Hoekstra M, Gerritsen J, de Jongste JC: **Respiratory symptoms in the first 7 years of life and birth weight at term: the PIAMA Birth Cohort.** *Am J Respir Crit Care Med* 2007, **175(10)**:1078-1085.
39. Zuidgeest MG, Koster ES, Maitland-van der Zee AH, Smit HA, Brunekreef B, Leufkens HG, Koppelman GH, Postma DS, de Jongste JC, Hoekstra MO: **Asthma therapy during the first 8 years of life: a PIAMA cohort study.** *J Asthma* 2010, **47(2)**:209-213.
40. Hollis S, Campbell F: **What is meant by intention to treat analysis? Survey of published randomised controlled trials.** *BMJ* 1999, **319(7211)**:670-674.
41. Campbell MK, Elbourne DR, Altman DG, CONSORT group: **CONSORT statement: extension to cluster randomised trials.** *BMJ* 2004, **328(7441)**:702-708.
42. Twisk J: *Applied Multilevel Analysis: A Practical Guide* Cambridge: Cambridge University Press 2006.
43. Martinez FD: **Development of wheezing disorders and asthma in preschool children.** *Pediatrics* 2002, **109(Suppl 2)**:362-367.
44. Morgan WJ, Stern DA, Sherrill DL, Guerra S, Holberg CJ, Guilbert TW, Taussig LM, Wright AL, Martinez FD: **Outcome of asthma and wheezing in**

the first 6 years of life: follow-up through adolescence. *Am J Respir Crit Care Med* 2005, **172(10)**:1253-1258.
45. Martinez FD: **What have we learned from the Tucson Children's Respiratory Study?** *Paediatr Respir Rev* 2002, **3(3)**:193-197.
46. Watts B: **Outpatient management of asthma in children age 5-11 years: guidelines for practice.** *J Am Acad Nurse Pract* 2009, **21(5)**:261-269.
47. National Asthma Education and Prevention Program (NAEPP): **Expert Panel Report. Guidelines for the Diagnosis and Management of Asthma: Update on Selected Topics.** *J Allergy Clin Immunol* 2002, **110(Suppl 5)**: S141-219.
48. Brunekreef B, Smit J, de Jongste J, Neijens H, Gerritsen J, Postma D, Aalberse R, Koopman L, Kerkhof M, Wilga A, van Strien R: **The prevention and incidence of asthma and mite allergy (PIAMA) birth cohort study: design and first results.** *Pediatr Allergy Immunol* 2002, **13(Suppl 15)**:55-60.
49. Horak E, Grässl G, Skladal D, Ulmer H: **Lung function and symptom perception in children with asthma and their parents.** *Pediatr Pulmonol* 2003, **35(1)**:23-28.
50. Luyt DK, Burton PR, Simpson H: **Epidemiological study of wheeze, doctor diagnosed asthma, and cough in preschool children in Leicestershire.** *BMJ* 1993, **306(6889)**:1386-1390.
51. Yoos HL, McMullen A: **Symptom perception and evaluation in childhood asthma.** *Nurs Res* 1999, **48(1)**:2-8.
52. Hederos CA, Hasselgren M, Hedlin G, Bornehag CG: **Comparison of clinically diagnosed asthma with parental assessment of children's asthma in a questionnaire.** *Pediatr Allergy Immunol* 2007, **18(2)**:135-141.
53. Hoek G, Wypij D, Brunekreef B: **Self-reporting versus parental reporting of acute respiratory symptoms of children and their relation to pulmonary function and air pollution.** *Int J Epidemiol* 1999, **28(2)**:293-299.
54. Pless CE, Pless IB: **How well they remember. The accuracy of parent reports.** *Arch Pediatr Adolesc Med* 1995, **149(5)**:553-558.
55. American Thoracic Society and European Respiratory Society: **ATS/ERS recommendations for standardized procedures for the online and offline measurement of exhaled lower respiratory nitric oxide and nasal nitric oxide.** *Am J Respir Crit Care Med* 2005, **171(8)**:912-930.
56. Nicole Beydon, ATS/ERS Working Group on Infants, Young Children Pulmonary Function Testing: **Interrupter resistance: What's feasible?** *Paediatric Respiratory Reviews* 2006, **7(Suppl 1)**:S5-S7.

Pre-publication history

The pre-publication history for this paper can be accessed here: http://www.biomedcentral.com/1471-2458/10/555/prepub

doi:10.1186/1471-2458-10-555
Cite this article as: Hafkamp-de Groen *et al.*: **Early detection and counselling intervention of asthma symptoms in preschool children: study design of a cluster randomised controlled trial.** *BMC Public Health* 2010 **10**:555.

Submit your next manuscript to BioMed Central and take full advantage of:

- **Convenient online submission**
- **Thorough peer review**
- **No space constraints or color figure charges**
- **Immediate publication on acceptance**
- **Inclusion in PubMed, CAS, Scopus and Google Scholar**
- **Research which is freely available for redistribution**

Submit your manuscript at
www.biomedcentral.com/submit

BioMed Central
