# Supplementary material for: Evaluation of Systematic Assessment of Asthma-Like Symptoms and Tobacco Smoke Exposure in Early Childhood by Well-Child Professionals: A Randomised Trial
Source: PLoS One. 2014 Mar 13;9(3):e90982. doi: 10.1371/journal.pone.0090982 (PMC3953324; doi:10.1371/journal.pone.0090982)
Supplement: Protocol S1 — Original Study Protocol. (PDF) [file pone.0090982.s005.pdf]

Study Protocol

Title: A randomized trial of early detection of asthma-related symptoms in pre-school children at preventive Child Health Centers in the Netherlands

Introduction

The procedure for early detection of asthma-related symptoms in pre-school children is based on medical history taking, in accordance with the GP-clinical guidelines [1-3]. Contrary to the common situation in the field of early detection, in this specific case there is -by definition- no "gold standard" (a test more refined and valid than the early detection procedure). Therefore, parameters for the performance like "sensitivity", "specificity", and "predictive value of a positive/negative test result" are not applicable. Success or failure of the intervention will be measured according to the extent to which the goals of the intervention will be achieved (see "Problem definition").

Goals of early detection at child health centers

Early detection (and management) has two distinct objectives:

(1) "Short-term" objective: Immediate higher generic and respiratory health-related quality of life during the age period 0-4 years;

(2) "Medium/long-term" objective: Reduced probability of doctor-diagnosed asthma (and higher health-related quality of life; better lung function parameters) in later life, which will be measured at age 5 years (follow-up at age 7 years will be prepared).

Informed consent

Informed consent will be obtained within the Generation-R framework. The parents will be informed and asked for consent during pregnancy at inclusion into the study. At the first Generation-R post-natal home visit additional information will be presented and consent for the study, including this trial, will be asked for. Approval of the Medical Ethical Committee will be applied for. Participation in the study is fully voluntarily.

Randomization

Randomization at the individual level is no suitable option as a physician who applies standardized early detection to half of the cohort members, can be expected to be more alert than usual with regard to children in the control group as well. So, randomization will be done at the level of the Child Health Centers (CHC's; n=30) within three strata of social-deprivation of the catchment area (index formulated by COS-Rotterdam). In order to reduce possible cross-over effects, instructions with regard to the study-intervention will be strict, yet organized with little publicity, and not assessable for those who are not employed by the centers where the intervention takes place.

Study intervention

Physicians at 15 "intervention-CHC's" (randomly chosen) will implement the intervention at age 14, 24, 36, and 46 months. The early detection instrument is a concise standardized medical history adapted from the ISAAC-asthma-core questionnaire (extensively used in the PIAMA birth cohort studies) and validated in an open population pre-school children [III]. Referral to the GP is indicated for children (not having asthma medication and not being monitored by the GP in the last 6 months) with the symptom-diagnosis "recurrent cough/wheezing/chest congestion". Operationalization during last year: (a) at least 5 (at age 14 months: 3) periods of minimally 10 days with cough/chest congestion; (b) and/or recurrent episodes of wheezing during or following viral infections. If children fit the referral criterion 5 additional items will be assessed as recommended in the guidelines (constitutional eczema; familiar predisposition; increase in wheezing/dyspnea with increasing age; signs of allergic sensitization; smoking/pets in the household).

History taking will be computer-assisted, as part of the automated client files for the Generation-R project. A referral letter, if indicated, will be drafted automatically. It will inform the GP according to the diagnostic format applied in the clinical guidelines. As a "courtesy" at the back of the letter a synopsis of the asthma guidelines for 0-4 year olds, drafted by the local GP-association, will be printed. Also a health information leaflet will be printed for the parents, in order to support the referral. The information in this leaflet will be automatically tailored to the situation in this family, based on the stored data (e.g. detected for the first time? passive smoking/pets in the household?). Computer-assisted diagnosis and computer-tailored health information improve validity and effectiveness (VIII).

Once detected, the monitoring and management of asthma-related symptoms will be performed mainly in primary care practices according to the guidelines [1], complemented by specialized care [2].

"Control" intervention
At 15 other CHC's the current routine practice will be applied: no active, standardized detection procedure. Of course, parents may spontaneously mention asthma-related symptoms or the physician may notice symptoms. So, fewer cases will be detected, on average longer time after the start of the symptoms than at the "intervention-CHC's". The referral/health education given to the parents generally will be less informative than at the "intervention-CHC's".

Short-term health outcomes: 0-4 years
Short-term outcomes refer to the improvement in quality of life measured at age 18, 30 and 42 months within the routine Generation-R measurements:
- Frequency of asthma-related symptoms-questionnaire (last 4 weeks/12 months) [ISAAC/PIAMA];
- Generic health-related quality of life of the child (last 4 weeks): Health Utilities Index 2/3; Child Health Questionnaire-Infant/Toddler;
- Additionally: frequency of GP visits outside office hours and emergency hospital visits with respiratory symptoms as reason for encounter (per 6 months during age 1-4 years).

The asthma-specific items are balanced in number with respect to the other symptom-questionnaires within Generation-R in order not to arouse specific attention, especially in the control-group. The results are blinded to the physicians. With regard to respiratory problems, "outside office hours"-medical consumption and emergency hospital visits are taken as proxy for better "control" over respiratory problems (less indicates better control). This will be inventoried automatically within Generation-R, given informed consent.

Medium-term health outcomes: age 5 years
At age 5 years asthma-related medical history is more evident than in pre-school children and may be complemented with blood tests for allergic sensitization (RAST). At this age advanced assessments like MicroRint (lung function) and exhaled NO (asthmatic airway inflammation), which a.o. have been tested in the PIAMA cohort, will become available in Generation-R. All measurements will be integrated in the Generation-R measurement at the school-physician office (GGD):
- Frequency of asthma-related symptoms-questionnaire [ISAAC/PIAMA], generic health-related quality of life (HUI2/3; CHQ-Parent Form [8-9]) and "outside office hours/emergency" care utilization for respiratory problems as defined above (measured in all children in both intervention/control group);
- The subgroup with respiratory symptoms at a level indicating asthma (maximally 20%) in both intervention/control group will be asked to complete a validated 4-week 6-item daily symptom score list [III];
- At least for this sub-group in both intervention/control group, also a multi-RAST/Phadiatop-test (blood samples by Generation-R) as indicator of allergic sensitization will be performed. From a parallel Generation-R-project in both intervention/control group probably a broader assessment of multi-RAST and/or specific-RAST (house dust mite/cat/dog) will be made available for this study;
- Lung function in all children will be assessed by micro-RINT as marker of asthma and atopy. This is a validated non-invasive method applicable to young children (the Sophia-EMCR group determined normal values of interrupter resistance: P.J.F.M. Merkus, J.C. de Jongste et al. Interrupter resistance in pre-school children; measurement characteristics and reference values. Am J Respir Crit Care Med 2001, in press). These data will come from a parallel Generation-R project;
- Exhaled nitric oxide (NO) in all children. This is a marker of asthmatic airway inflammation that can be applied non-invasively to young children. Exhaled air is collected in a balloon under standardized conditions, and its NO content measured later (Q. Jöbsis et al. Off-line sampling of exhaled air for nitric oxide measurements in children. Eur Respir, in press). These data come from a parallel Generation-R project;
- By way of automated decision rules, in accordance with the clinical guidelines, in both intervention/control group all data will be reviewed and the presence/absence of asthma at 5 years will be established. Independently, also an expert-panel (GP, pediatrician and pediatrician/pulmonologist) will, unaware of assignment to intervention/control group, evaluate all child-data (except for clear cases) and assign doctor-diagnosed asthma or not. Reported symptoms at earlier ages and familiar predisposition (available for both groups) will be taken into account.

Outcomes at 7 years and later
At age 7 years it is considered that asthma can be diagnosed with sufficient certainty, when the foregoing can be complemented with usual lung function measurements (e.g. 4-week peak-flow diary). In study year 6 a proposal will be submitted for outcome measurement at age 7. In the future,

within Generation-R, outcomes at older ages, even into adulthood, may be measured.

Additional variables

The season of data collection and co-morbidity, like constitutional eczema and allergies will be recorded in Generation-R. Data on prescribed (asthma) medication and medical care utilization (respiratory symptoms and other reasons for encounter) will be gathered via automated procedures within Generation-R from the pharmacists, GP's and hospitals, given informed consent. In referred children (/parents) the compliance to the referral and the course of action recommended by the GP will be inventoried. A random sample of 120 referred children (/parents) will be interviewed (semi-structured) 6 months after referral, to evaluate reported advantages/disadvantages of and satisfaction with the early detection and follow-up. Ten CHC-physicians and 25 GP's (random samples) will be asked for semi-structured interviews on their experiences/opinions.

Power analysis

Net 5000 children are included at birth in both intervention/control group; at age 14 months resulting in at least 4750 per group. Minimally 3325 children per group will participate in outcome measurement at 5 years (70%; see "Feasibility"). Taking into account cluster randomization, assuming a prevalence of asthma of .05 in the control group at age 5 years, alpha .05 and a power of .80, an absolute difference in the proportion of children with asthma between intervention/control group of .02 can be established with a total of 30 CHC's/9500 children starting in the study at 14 age months.

Statistical analysis

A multi-level analysis applies in order to allow for dependency between the individual measurements within the 30 randomized CHC's. The main outcome (yes/no asthma at 5 years) will be analyzed by means of logistic regression analysis with independent variables: intervention/control group, season, genetic predisposition, smoking, pets, co-morbidity (eczema, allergy), age/sex. Complementary subgroup analyses will be done for gender, parental educational level (high/low) and ethnic background.

The impact of the early detection-intervention on asthma-related care utilization (including use of prescribed drugs) will be described and compared with the control group by means of multiple linear or logistic regression analysis (depending on the outcome variable). Estimates of direct medical costs (e.g. GP-visits, prescribed drugs) will be made. (Dis)advantages/satisfaction with the early detection/follow-up, reported by parents/professionals, will be described.

References

1. NHG Standaard astma bij kinderen (eerste herziening). Huisarts en Wetenschap 1998 42(3):130-143.
2. Hoekstra MO. Behandeling van astma bij kinderen: herziene richtlijnen van kinderlongartsen. NTVG 1997 141(6):2223-2229.
3. Jones A. Screening for asthma in children. Br J Gen Pract 1994 44(381):179-183.
